# Health Professionals’ Experience with the First Implementation of the Organizational Health Literacy Self-Assessment Tool for Primary Care (OHL Self-AsseT)—A Qualitative Reflexive Thematic Analysis

**DOI:** 10.3390/ijerph192315916

**Published:** 2022-11-29

**Authors:** Natascha Stuermer, Saskia Maria De Gani, Anna-Sophia Beese, Jennifer Giovanoli Evack, Rebecca Jaks, Dunja Nicca

**Affiliations:** 1 Epidemiology, Biostatistics and Prevention Institute, University of Zurich, 8001 Zurich, Switzerland; 2 Center for Health Literacy, Careum Foundation, 8032 Zurich, Switzerland; 3 Careum School of Health, Kalaidos University of Applied Sciences, 8006 Zurich, Switzerland

**Keywords:** organizational health literacy, implementation, Normalization Process Theory, self-assessment, primary care setting, reflexive thematic analysis

## Abstract

Organizational health literacy (OHL) is crucial for public health, in turn health care organizations play vital roles in improving populations’ health literacy. Therefore, the aim of this qualitative study was to explore how the organizational health literacy self-assessment tool (OHL Self-AsseT) was implemented, used, and understood by primary care teams from a network of general practices and a Home Care Service Organization in Zurich, Switzerland. Reflexive thematic analysis with a constructivist orientation was used to analyze data from 19 interviews pre- and post-OHL Self-AsseT use. Normalization Process Theory supported structuring of inductively developed themes. Findings show that the participants experienced working with the OHL Self-AsseT meaningful, as it helped with “Addressing OHL construction sites” so that they could “build momentum for change”. The experience of “Succeeding together in construction” led to a “feeling of team-efficacy during change”. Practical use of the tool and/or discussions about OHL led to a growing conceptual understanding, which was described as “Using a construction plan–making sense of ongoing OHL activities”. To conclude, the OHL Self-AsseT encouraged teams to initiate change, led to greater team-efficacy and supported the construction of OHL. Improved implementation strategies will support this intervention’s scale-up as a base for effectiveness testing.

## 1. Introduction

Organizational health literacy (OHL) is vital for public health. In addition to increasing the health system’s responsiveness to patients’ needs, it can impact individual health literacy and improve population-level health outcomes [1,2]. Unfortunately, the numerous definitions of OHL have made it difficult to construct a unifying conceptual model. Therefore, for a concise description, we looked at the review of Sørensen et al. [3], which noted that “a shared characteristic of these definitions is their focus on individual skills to obtain, process and understand health information and services necessary to make appropriate health decisions.” Using these common actions as defining criteria, we have concluded that a health-literate health care organization is one that enables its care workers—and, through them, its clients—to find, understand, and use health information and services [4,5]. In contrast, health literacy is the degree to which individuals are able to find, understand, assess and apply health-related information [3].

The task of building health literacy is neither simple nor small. Over half of the people who live in Europe have difficulties dealing with health information and services [6]. The associated costs—both human and economic—are high: people with lower levels of health literacy tend to engage less in health-promoting behaviors, feel generally less healthy and use their health systems more often [6,7,8].

At the same time, even as health care organizations are providing increasingly complex services, few are adequately addressing and strengthening their patients’ health literacy. A recent scoping review of 18 descriptive studies showed that, as health professionals tend to have a limited understanding of health literacy, they also tend not to implement recommended practices, e.g., using plain language or working to empower patients in their daily practice [9]. Interventions for health care organizations to improve their clients’ health literacy must specifically address their systems’ responsiveness to individuals’ health literacy needs. In this respect, adequately-informed health care organizations and practitioners can play central roles [9,10].

Over the past decade a limited range of instruments have been developed to measure and improve OHL. A recent scoping review [5] found seventeen that measure various OHL dimensions. To date, however, most evaluations of these instruments have generally focused either on descriptive feasibility or case-studies; few have included rigorous pre-post-evaluation designs. Moreover, the described instruments differ regarding both structure and content. Some consist entirely of OHL assessments, while others include assessment and improvement measures. Likewise, regarding focus, some examine single dimensions, whereas others are multidimensional [5,9]. Bremer et al. 2021 review mapped six main dimensions relevant for multidimensional OHL assessment and improvement measures: (1) communication with service users; (2) easy access & navigation; (3) integration & prioritization of OHL; (4) assessments & organizational development; (5) engagement & support of service users and (6) information & qualification of staff.

The year before that review was published, in response to the shortage of rigorously evaluated OHL instruments, especially in primary care settings, our research group developed the “Organizational Health Literacy Self-Assessment Tool for Primary Care” (OHL Self-AsseT). Its development was based on available evidence as the Vienna Concept of Health-Literate Hospitals and Healthcare Organizations (V-HL0-1) [11] and a systematic process described by De Gani et al. [12]. This multidimensional instrument aims to enable primary care teams to first assess their OHL across six dimensions and then plan and implement improvement measures. The dimensions are: (1) provide easy access to and facilitate navigation within primary care services; (2) communicate in plain and easy to understand language; (3) promote users’ health literacy; (4) promote staff members’ health; (5) incorporate health literacy into the management and organizational structure and (6) promote health literacy at care interfaces, networks, and further activities of the organization. Five of these six defined dimensions are comparable to those described by Bremer et al. [5]. Basically, our version combines Bremer’s [5] dimensions 4 and 5 and adds care interfaces in dimension 6. We included this dimension because patients in primary care settings often have a variety of care providers: At patients’ interfaces with those providers, health literacy is particularly important to improve coordination of care.

Health professionals in primary care operate as first point of contact and provide continuity of care.

The OHL Self-AsseT development was planned as part of a stepwise evaluation and scaling process within Swiss primary care. Initial steps included the first implementation, use and evaluation of the OHL Self-AsseT in a small but scalable unit [13]. The smallest scalable unit was initially defined as general practitioners’ practices (GPPs) of a larger network and nursing teams of a large home care service organization (HCSO) in the Canton of Zurich, Switzerland. Understanding how the OHL-Self AsseT is implemented and used by health care professionals in the complex primary care context is important for its further development. The Medical Research Council updated framework for evaluating complex interventions states that to achieve an intervention’s intended outcomes, evaluation data from the first implementation process should be used to guide both (a) the advancement of the innovation—in this case, the OHL Self-AsseT—and (b) the improvement of implementation strategies to support the instrument’s use in real-life care settings [14]. Accordingly, before larger implementation or effectiveness evaluation, we will use evaluation data for such improvements [13]. Conducted in this way, implementation is a continuous, adaptive mechanism towards normalization of new practices [15]—in this case, evidence-based OHL practices. As an early step in this process, the aim of the qualitative study reported here was to explore how primary care professionals implemented and experienced the use of the OHL Self-AsseT as teams.

## 2. Methods

### 2.1. Design

**Figure 1:**
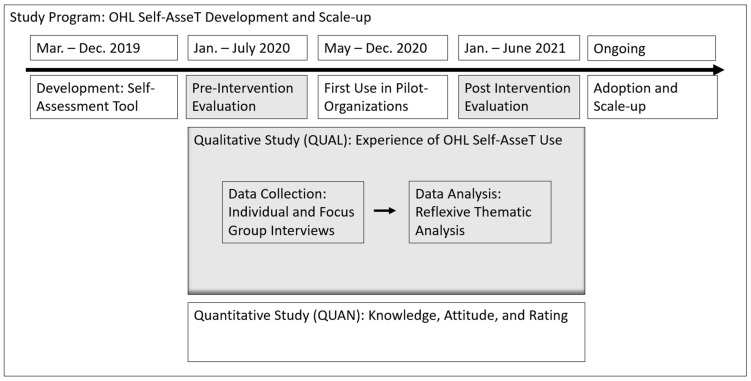
Embedded qualitative study design adapted from Creswell and Planto Clark, 2017 [15].

This qualitative study is embedded in a larger mixed-method study program [16] that aims to evaluate, advance and scale the OHL Self-AsseT in Swiss primary care settings. Before and after the first use of the OHL Self-AsseT, qualitative data on expectations and experiences with the OHL Self-AsseT were collected and analyzed from individuals who had agreed to use and apply the tool and/or enabled its use within their organizations (illustrated in Figure 1, below). In parallel, quantitative data on OHL knowledge, attitudes, and ratings were collected and analyzed. The results of those analyses are presented elsewhere [17].

For the embedded qualitative study, to gain insights into primary care professionals’ expectations and experiences with the use of the OHL Self-AsseT, we applied a reflexive thematic analysis [18]. This allowed the inductive identification and interpretation of themes based on methodological orientation. To do so, we used a constructivist orientation based on the assumption that knowledge and meaning are constructed through continuous iterative processes between what is already known and current experiences [19].

Later in the process of data analysis, we structured the inductively developed themes using the four constructs of Normalization Process Theory (NPT). The constructs highlight key mechanisms that influence the normalization of new practices: (1) *Coherence*, i.e., the extent to which participants can a make sense of an intervention; (2) *Cognitive Participation*, i.e., how the participants adopt the intervention; (3) *Collective Action*, i.e., the participants’ operational work and (4) *Reflexive Monitoring*, i.e., the participants’ assessments of how the new practice affects them and their work [15,20,21,22].

This study was conducted and reported in accordance with the Consolidated Criteria for Reporting Qualitative Research [23].

### 2.2. Setting and Recruitment for Intervention

The OHL Self-AsseT was used in two primary care organizations in the Canton of Zürich, Switzerland: a network of GPPs and a large HCSO. Both had previously participated in the development of the OHL Self-AsseT. The network of GPPs consisted of 62 practices whose main service is primary medical care. These practices employ medical doctors and medical practice assistants (MPAs, i.e., clinic administrators with training to perform common medical procedures such as blood collection or injections). The HCSO has nine locations, covering 56 nursing teams, which consist of registered nurses, nursing assistants and advanced practice nurses (APNs, master’s degree prepared nurses). Their main services are nursing care and housekeeping services at the patients’ homes.

To start with the OHL Self-AsseT, a managerial person in each organization recruited teams who were willing to implement the tool. Eight HCSO and four GPP teams agreed to participate. Before the implementation started, two teams from the HCSO declined to participate due to sudden reductions of their personnel resources. Therefore, six HCSO teams and four GPP teams used the OHL Self-AsseT.

### 2.3. Intervention and Implementation Support

The OHL Self-AsseT is an intervention that supports primary care practice teams to assess their OHL across six dimensions, as well as to plan and implement improvement measures. It consists of three modules: (1) a manual with instructions on how to use the OHL Self-AsseT as a team within an organization; (2) a checklist for the self-assessment of OHL across six dimensions and (3) a handbook to support planning and implementation of improvement measures. As laid out in the instruction manual, each participating team was asked (a) to build a self-assessment team of at least two individuals from different health professions and hierarchical levels and (b) to name a coordinating person to be responsible for the coordination of the self-assessment process. Then, the coordinating person for each team received detailed information about their role, project procedures, and materials from the research team. They persons then coordinated the intervention process along the five steps documented in the instruction manual: (1) After every member of the self-assessment team had filled in the checklist individually, all team members discussed their individual ratings and reached a consensus for the overall team rating; (2) based on this consensus, the team identified needs for action and (3) defined one to three goals for OHL improvement. (4) Subsequently they discussed and defined one or two improvement measures per goal and finally, (5) the planned measures, which were (ideally) implemented by the whole team. It was recommended that the planned measures be regularly reviewed. The intervention was carried out in each participating team’s respective location. The full time required for the intervention depended on the organizational circumstances: on average it took 2–3 months. Implementation strategies, i.e., techniques to enhance the adoption and implementation of the OHL Self-AsseT intervention included: (1) regular monthly online meetings between our research team’s project manager and the coordinating persons from the self-assessment teams (comparable to project champions) to answer ongoing questions and support timely implementation of all steps; (2) nomination of one contact person (managerial person) each from the GPPs’ and the HCSOs’ respective organization management to answer questions (leadership support) [24,25]. Basically, the implementation support was planned the same for all, in the results we described commonalities and differences in adoption.

### 2.4. Sample for Evaluation

To qualitatively evaluate each implementation of the OHL Self-AsseT, we used a purposive sampling approach that required a maximum variation sample of 15–20 individuals [26]. The inclusion criterion for study participation was to be in a leadership position within this project and/or one of the participating organizations. To achieve variation regarding experiences, we recruited participants based on the following criteria: members from both participating organizations and each team (e.g., HSCO, GPPs); from a variety of professions (e.g., medical doctors, MPAs, registered nurses, nursing assistants); performing different functions within the project (e.g., coordinating persons, managerial persons responsible for this project); and employed at diverse hierarchical levels within their organizations (e.g., manager position, employee). Table 1 provides an overview of the interview participant sample.

### 2.5. Data Collection

Data were collected pre- and post-use of the OHL Self-AsseT. Overall, 19 professionals participated, of whom five participated in both pre- and post-intervention interviews. Six individual interviews were conducted in the pre-intervention phase between March and June 2020; the post-intervention phase included three focus group interviews with, respectively two, three and five participants each (n = 10 participants). Ten further individual interviews were conducted between October 2020 and April 2021 (see Table 1). All participants received written study information beforehand and gave informed consent.

Interview guides with open-ended questions were developed and used in all interviews. For the pre-intervention data collection, this included questions on participants’ perceptions of OHL, the project itself and their motivation for participating, as well as their expectations regarding the tool. For post-intervention interviews, the guide included questions on the use of the OHL Self-AsseT, the interactions and leadership during the process, and changes experienced due to the intervention. Two focus group moderators stimulated discussion on how the changes the intervention had sparked were reflected in daily practice, while ensuring that all participants were able to contribute their perceptions and experiences. Due to the COVID-19 pandemic, all interviews were conducted over a video conferencing system. All were conducted in German or Swiss-German. Focus group discussions lasted 50–80 min, individual interviews 15–60 min (mean: 30 min). Data were video/audio recorded and transcribed verbatim.

To understand the performance and actions of the self-assessment teams implementing the OHL Self AsseT, we needed minimal quantitative data. We used the ratings per dimension from the checklist (module 2), the full checklist is published by De Gani et al. [12]. The collected data from the intervention documentation (i.e., needs for action and planned improvement measures) is exemplary. The full quantitative results are presented elsewhere [17]. Our goal was to describe the participating team’s performance in relation to emerging qualitative themes in ways that would improve our understanding of the implementation process.

### 2.6. Data Analysis

Intervention documents were subjected to descriptive summative analysis. For the ratings of the checklist a descriptive analysis of the participating teams was conducted. The values ranged from 0%, indicating no fulfillment of the corresponding criteria and 100%, indicating that the criteria were completely fulfilled by the organization, according to the self- assessment of the participating teams.

Qualitative interview data were analyzed using the inductive approach of reflexive thematic analysis supported by the MAXQDA, (VERBI Software, Berlin, Germany) software package [27]. The dataset incorporates pre and post data, which were analyzed as a whole. To ensure credibility, the six iterative phases were conducted by the research group using an active discursive process (see Table 2). Data were analyzed and interpreted (rather than simply paraphrased or described), always following the research question and integrating Braun and Clarke’s 15-point checklist for reflexive thematic analysis [28]. This method allows the use of theories.

## 3. Results

**Figure 2:**
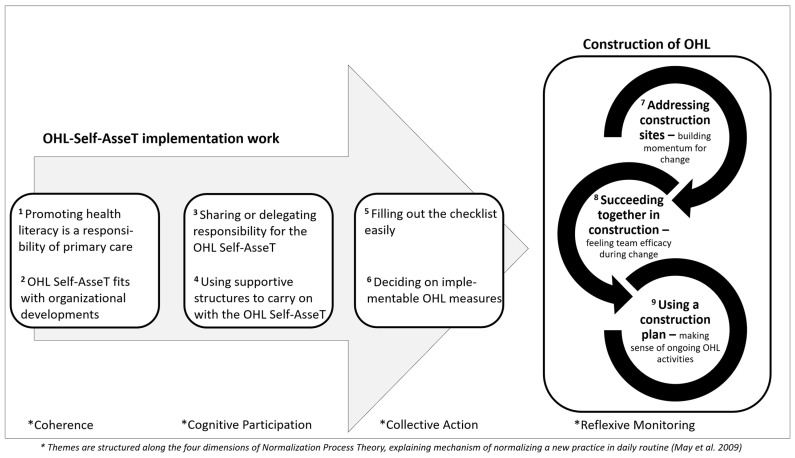
Thematic map: OHL Self-AsseT implementation work leading to construction of OHL in primary care settings.

The participants who shared their OHL Self-AsseT experiences were predominantly female, with ages ranging from 20–65; most were in their forties. Both their professional backgrounds and their positions in their organization were diverse. At the time of the interview, all had worked for several years in their respective primary care settings (9 in HSCOs, 10 in GPPs) and were actively involved in the implementation of the OHL Self-AsseT by supporting the project, or conducting assessments and planning improvement measures. Their stories describing their implementation work—laying the foundations of OHL construction in their practice fields—are reported in a thematic map in relation to eight themes, which are structured along the four NPT dimensions (coherence, cognitive participation, collective action, reflexive monitoring) (Figure 2). The following paragraphs present an overview of that thematic map, followed by an in-depth description of the themes, accounting for commonalities and differences in the participants’ experiences.

### 3.1. Overview of Thematic Map: OHL Self-AsseT Implementation Work Leading to the Construction of OHL in Primary Care Settings

The thematic map shows the implementation work which that leads to the construction of OHL as it relates to the first five themes. It starts with two themes dealing with why health professionals participated in the project and were motivated to use the tool (Coherence): first, Promoting *HL is a responsibility of primary care*; and second, *OHL Self-AsseT fits with organizational developments*. To work with the OHL Self-AsseT, the teams adapted the process described in the intervention instructions to the conditions of their setting (Cognitive Participation). The third theme *Sharing or delegating responsibility for the OHL Self-AsseT* highlights the main adaptations; and the fourth, *Using supportive structures to carry on with the OHL Self-AsseT*, describes the factors that supported the implementation process’s continuation. All teams were able to conduct all steps of the OHL Self-AsseT (Collective Action). The specific actions they performed are described in theme five: *Filling out the checklist easily* and six: *Deciding on implementable OHL measures*.

Three additional interconnected themes describe the changes participants experienced following implementation of the OHL Self-Asset, and their reflections on its use, i.e., Reflexive Monitoring. These are based on the metaphor of “construction.” Several participants used this metaphor to describe their work on OHL problems, which they called ‘construction sites.’ Accordingly, theme seven, *Addressing construction sites—building momentum for change* describes how participants used the OHL Self-AsseT to initiate meaningful changes. Theme eight, *Succeeding together in construction—feeling team efficacy during change* highlights the experience of participants who discussed ‘construction sites’ as a team and the empowering effect these discussions had on their work toward improved OHL. Approaching OHL implementation from the perspective of a construction team led to a sense of shared success, fostering a justifiable sense of group efficacy. Theme nine, *Using a construction plan—making sense of ongoing OHL activities* covers the intervention users’ development of an extended conceptual understanding of OHL, which positively redefines several everyday activities. This understanding is comparable to a construction plan: it lays out connected tasks in ways that help users both monitor and ensure progress.

### 3.2. Coherence: Why People Engaged in the Project

Two themes cover the question of why the interviewees and their teams considered it meaningful to participate in the intervention and engage themselves in the process: *Promoting health literacy is a responsibility of primary care*; and *OHL Self-AsseT fits with organizational developments*.

#### 3.2.1. Promoting Health Literacy Is a Responsibility of Primary Care

Participants stated that they considered health literacy meaningful and important because of its relevance to their work with patients. However, in the evaluation of what the health literacy part of their work includes, explanations varied considerably between individual health professionals both of the GPPs and the HCSO teams.

One perception all participants shared was that, as health professionals in primary care settings, they are directly responsible for improving their patients’ health literacy. Some explained that before participating in this project they did not use the term health literacy, but had frequently applied individual aspects of the concept to their practice. As health professionals, they considered themselves front-line providers of reliable patient-oriented evidence-based information that supports patients’ decision making. One health professional described the importance of information provision from health professionals in contrast to other sources of information:

*“If we don‘t provide information, who will? The media or Google or such-like just don’t meet the need, as we as experts are available.”*

(I. MP4, Z. 798-802)

Another participant highlighted information provision regarding patients’ decision-making as a basic component of primary care:

*“It is important to me that we inform the patient well. And that they can decide for themselves. That’s a part of the empowerment. I have never wanted it any other way.”*

(I.F1, Z.580-587)

While providing patients with information was commonly described as an important health literacy-related responsibility, other responsibilities were explained with greater variety regarding content. Even professionals from the same organization or team appeared to have widely different ideas or priorities as to what tasks and responsibilities OHL entails—and thus, why participating in the project made sense. For example, whereas some participants predominantly mentioned the importance of health promotion activities (e.g., exercise, diet) for patients, others focused strongly on expectations regarding staff development through a better understanding of OHL. As one of the professionals stated:

*“I think… [the main value of exploring OHL] is really the enrichment in the job, the broadening and the increase in knowledge.”*

(I.FS1, Z. 444-446)

#### 3.2.2. OHL Self-AsseT Fits with Organizational Developments

The participants considered the project meaningful largely because it fits well with organizational development goals and processes focusing on quality improvement. Many also assessed the intervention as workable partly because they perceived openness to change in their organizations and partly because they considered the use of the tool as “pragmatic”.

HCSO participants noted similarities between the tool and their current organizational development processes. At the time of data collection, they were undergoing a reorganization process from nursing teams to self-organized teams, meaning there were no formal leadership positions; and teams had to organize their core tasks for themselves. This high-autonomy leadership approach, which is derived from ‘Buurtzorg models,’ promises high-quality home care [29]. Some participants envisioned the OHL project serving to help them fulfill their responsibilities both as individual employees and as teams. As one manager observed,

*“The self-check fits well with self-organization. There are indicators in it that are important for us, for example, empowering patients and empowering teams.”*

(I. FS1, Z. 660-663)

Participants from the GPP team emphasized their ambition to continuously improve quality of care within their organizations. As they expected the OHL Self-AsseT to yield useful results, they considered it worthwhile to participate in its testing, as one participant explained:

*“We already have had many information materials for patients from the medical network. And then we were EQUAM-certified (external quality promotion in ambulatory medicine) just last year. And we actually assumed a bit that our practice is not badly positioned.”*

(I.MP3, Z. 150-154)

Participants of both organizations emphasized the importance of the project being “*suitable for everyday use*”, i.e., not adding to the complexity of daily routines, but providing practical benefits. One-time use of the OHL Self-AsseT and the recommended improvement procedures seemed workable; and the expected benefits were in line with a general openness to change in both organizations. A coordination person described her reasons for agreeing to participate as follows:

*“It is a good chance—a view from the outside that gives us a chance to understand [our work] differently. And to look at points that we wouldn’t have [noticed]. And that was the decisive factor for me to participate.”*

(I.S5, Z.823-827)

### 3.3. Cognitive Participation: How Users Adapted the OHL Self-AsseT

The OHL Self-AsseT instructions specify that the self-assessment should be performed in a small group (a self-assessment team) consisting of at least two people from different professional groups and/or hierarchical positions. The composition of the self-assessment teams in relation to the overall teams (GPPs/HSCO) is presented in Table 3. Regarding adherence to the instructions, instead of forming a multi-professional group, one GPP team was composed entirely of MPAs. Additionally, regarding self-assessment team composition, the HCSO self-assessment teams included every team member, not sub-groups as recommended. This made the HCSO self-assessment teams larger than suggested. Additionally, whereas the coordinating persons in the HCSO varied regarding professional background, every GP team’s coordinating person was an MPA.

These (more- or less-) diversely-composed self- assessment teams described the adoption of the intervention in terms of two themes; *Sharing or delegating responsibility for the OHL Self-AsseT;* and *Using supportive structures to carry on with the OHL Self-AsseT*.

#### 3.3.1. Sharing or Delegating Responsibility for the OHL Self-AsseT

Participants described various strategies for adapting the composition of the self-assessment teams and their performance of the intervention fit their institutions’ possibilities and situations. The adaptations were shaped by the teams’ everyday methods of organizing themselves. The main difference was the sharing or delegation of responsibility for the OHL Self-AsseT. More specific variation was between sharing responsibility between team members versus delegating it to certain individuals.

HCSO participants decided in consensus that the self-assessment should be filled out by the entire team (5–14 persons). After all, as they had just organized themselves in equal teams, they considered it important both to involve everyone in the project and to share the responsibility. One manager recalled how members of one team first reacted to the project offer, then later informed her about a necessary adaptation:

*“At the first presentation this team said ‘oh yes, we’ll do it’ and when the coordinating person realized that it was too much for her alone, she simply got a team colleague and informed me (managerial person) that there are now two of them responsible for the coordination of the process. They just said: ‘We share the load’.”*

(I. S6, Z. 1268-1290)

The GPP participants described more variation regarding both self-assessment team composition and the application of the intervention. Whereas in some practices the self-assessment and planning of improvement measures were carried out jointly by MPAs and medical doctors (as recommended by the project requirements), in other practices this was only done by one or more MPAs. In one self-assessment team, the MPA filled in the intervention documents and the medical doctors were only involved to review the finalized documents (assessment and planning of improvement measures) without a team discussion. The interviewed medical doctors described that they did not invest more in the project due to their shortage of time and their usual division and/or delegation of tasks, but emphasized the trust between these two professions, as a medical doctor explained:

*“We give our head MPA free rein. So she takes on many organizational tasks … therefore, for me it [the intervention] was more an input that stimulated awareness [of health literacy].”*

(I.F1, Z. 75-78 and 20-23)

#### 3.3.2. Using Supportive Structures to Carry on with the OHL Self-AsseT

The participants reported using implementation support (e.g., reminders and meetings) to implement the OHL Self-AsseT. Because the OHL Self-AsseT was developed for independent use by organizations, a small degree of support had been built in by the research team. Reminders and a sense of accountability, as well as ‘*general reassurance regarding being on track*’ were important for all participants. Some teams also reported needs for implementation support, e.g., for ‘*leading the group*’ or to ‘*involve and motivate colleagues*’.

All coordinating persons reported that the informational support and guidance, e.g., regular online meetings and telephone contacts with the project manager of the research team, were either beneficial or vital. The availability of such contact left them feeling better-equipped both to prioritize the intervention in their everyday situation and to clarify questions of content. Additionally, a ‘*general reassurance of being on track*’—accompanied by reminders regarding milestones in the implementation process—were important for everyone. In example a participant stated:

*“It was a little difficult for us to find the time to perform the measures. But the project manager was very patient with us and gave us clear instructions and led us on towards the goal.”*

(I. S3, Z.131-135)

In the HCSO, coordination of the intervention was taken on by people with no former leadership experience. Therefore, they reported feeling insecure sometimes. The option to request support from a managerial person in ‘*leading the group*’ was described as beneficial by one team. Several HCSO participants reported having difficulties with the documentation and submission of them to the research team’s project manager, but also reported receiving support from the project manager or managerial person. One coordinating person from the HCSO described this as follows:

*“At the beginning I was also very unsure how it would work. But both the managerial person and the project manager supported us. They were always there and always available.”*

(I. S4, Z. 298-300)

In contrast, all of the GPP group’s coordinating persons held formal leadership positions. They explained that they had neither requested nor needed any support within their organization. Their main struggle was with ‘*the involvement and motivation of colleagues*’; consequently, they had to take on a considerable amount of extra work themselves to compensate as a participant stated:

*“Yes, it was difficult to get the motivation. I also said that they don’t have to do most of it. Just fill out the checklist and we’ll look at the measures together.”*

(I. MP3 Z. 281-283)

### 3.4. Collective Action: What Did They Do

The actions carried-out to implement the OHL Self-AsseT are described by two themes: *filling out the checklist easily* and *deciding on implementable measures*. This is based on data from qualitative interviews, ratings of the checklist and intervention-documentation.

#### 3.4.1. Filling out the Checklist Easily

The participants noted that they could easily fill out the checklists and rate how well their team/organization met the criteria of a health literate health care organization.

Most self-assessment teams had a meeting of one to two hours where they discussed their individual checklist ratings and reached a consensus checklist rating as to whether they (rather) agreed or (rather) disagreed with each of the checklist’s 75 item statements. They mentioned that they had no difficulties in filling out the checklist. However, different perspectives on how to answer items were described, especially between MPAs and medical doctors. They explained that in the group, they had to reflect about the item’s meaning and discuss their organizations performance. Through an understanding of different perspectives and a consensus process in the self-assessment teams, they experienced a broader awareness and learning in respect to their organizations HL. One participant explained this as follows:

*“we had to think a little bit about how much we know about the item or how that runs in the organization. We were not aware of certain topics beforehand.”*

(IS2, Z.568-572)

After the computer-assisted entry, participants received a score in percentage, per dimension, calculated by the software (Microsoft Excel, Microsoft Corporation, Redmond, WA, USA), representing the level of OHL. The self-assessment of the OHL level per dimension varied between 59.6–75.3% between teams (see Table 4). Dimension 4 ” Promoting health literacy of staff members “ was rated lowest, both by HCSO teams and GPP teams (59.5%). Detailed data on OHL level scores are presented in Beese et al. (under review) [17].

The participants felt, that the summarized scores presented as percentages and visually with bar-charts and radar charts matched their perception and expectations. They also rated their level of OHL as “satisfying” or as “passing the self-assessment”. Although they also mentioned some unexpected results, that served as eye openers, as one participant of a GPP remembered:

*“And we actually assumed a bit that our practice is not badly positioned. Nevertheless, we found afterwards quite many points, that we could improve.”*

(I.MP4, Z. 23-26)

#### 3.4.2. Deciding on Implementable OHL Measures

Based on the OHL levels, the self-assessment teams brainstormed needs for action and preliminary goals for OHL improvement. Followed by setting priorities in respect to which OHL improvement goals they wanted to pursue. Then, they decided on improvement measures to achieve the goals they had defined. Interestingly, the self-assessment teams planned goals and improvement measures not only for dimensions with lower OHL levels, but also for dimensions they had rated higher. They considered these improvement measures as very feasible and/or with a great potential for change.

The planning of improvement measures was supported by a separate document. For this step, they also had a specific handbook [12] with information and tools to plan measures. However, the participants stated that they did not use the handbook. They explained that the preceding step made it clear which measures they needed to implement. One participant stated:

*“I had the impression that the setting of measures usually happened very naturally in the team. Finding the problem usually took a little time, because we had to come to a common ground. The measures then just flowed out.”*

(I.S5, Z. 645-651)

Table 4 gives an overview of the OHL assessment per dimension with one example each of planned goals and measures. Most measures were planned for Dimension 1 *Provide easy access to primary care service and facilitate navigation within* and Dimension 4 *Promoting health literacy of staff members*. These measures varied from creating informational brochures for patients, organizing training and additional education on health literacy for colleagues to improving workflow and communication.

In addition to the officially documented measures, participants reported in the interviews that they had been inspired by the project to pursue other measures which they had not recorded in the official intervention documentation. One GPP team, for example, began calling their patients more actively after hospitalization. This corresponds to a measure in Dimension 6. The practice’s team reported that the patients acknowledged and appreciated the concern. Another participant mentioned the following example:

*“What we did in the team was pay more attention to our own health. For me, that was an additional goal, on my part. And we really did pay more attention to it.”*

(I.FP02, Z.73.76)

### 3.5. Reflexive Monitoring: What Do They Think about the Project

The qualitative post-evaluation data described the changes experienced by the participants due to their use of the OHL Self-AsseT in three themes: *Addressing construction sites—building momentum for change*; *Succeeding together in construction—feeling team efficacy during change* and *Using a construction plan—making sense of ongoing OHL activities*.

#### 3.5.1. Addressing Construction Sites—Building Momentum for Change

The participants described how they received the stimulus and the structure to make changes by using the OHL Self-AsseT. One explained this metaphorically as ‘addressing construction sites within an organization’. Similar language was used by others to explain what had happened due to the intervention. These experiences were similar among both GPP and HCSO personnel.

The participants reported that they were motivated to establish improvement measures because they had seen their OHL ratings visually, noting in particular that, while some dimensions already showed high approval levels, others did not. Moreover, the participants rated the checklist as a comprehensible and useful sense-making instrument: mostly, the items were easily understood. They especially valued the readability of the assessment graphics, as one participant stated:

*“It is clear, transparent, you see at a glance where we are strong, where our weaknesses lie. It is easy to use.…But everything you need is included. It is well structured.”*

(MP1, Z. 1045-1047)

Participants described working with the tool as sense-making. They explained that the checklist rating outcomes reminded them of problem areas or ‘construction sites’ that they had long wanted to address and that were also important for their patients. However, during their intensive everyday workload, they had never found the time—or set the priority—to address the issues. The OHL Self-AsseT finally provided both the impulse and the structure to do so. A participant from the GPPs describes such a long-standing issue they have finally successfully tackled:

*“The patients always had to ask where the toilet is, although there was a sign, but the sign wasn’t very good. And that is now a measure, where we changed something, we made the sign bigger and we noticed after a very short time: the patients no longer have to ask, the sign is clear. So it’s super. We knew actually that we should have changed that a long time ago, but now, thanks to the pilot project, we really did undertake the change.”*

(I. MP1, Z. 80-88)

Simultaneously, the participants described how working with the tool had helped them to recognize other problematic areas that had not been in the foreground or in their minds at all. Some teams planned to implement straightforward improvement measures; others found that the joint reflection session stimulated them to come up with creative new ideas on how to improve problematic areas. The HCSO managerial person explained this innovative spirit as follows:

*“And great things have come out of the project. In one team, the need for action was to make the HCSO more visible to the outside world. They were able to use a showcase (storefront) at their location for free, where they can advertise for the HCSO, also on various topics. And there they also want to bring in health-promoting things that also strengthen health literacy. I have never seen anything like this in my career, that a team has simply done this on its own. So I attribute that to the project.”*

(S.5, Z. 155-166)

#### 3.5.2. Succeeding Together in Construction—Feeling Team Efficacy during Change

Participants experienced that exchanging, reflecting on, and addressing the ‘construction sites’ with their groups strengthened their teamwork, feeling successful in the implementation of improvement measurements. When interaction within a team was lacking, the process was perceived as exhausting. Within the teams, the combination of collective construction with experiences of success generally led to feelings of efficacy.

Participants who had experienced such exchanges emphasized that, along with the success of the initiated measures, the sense of sharing in progress left a positive memory and had lasting positive effects on teamwork. As one HCSO coordinating person remembered,

*“we really worked together as a team on a project and somehow reached our goal in the end. That was also enriching. It was a beautiful experience.”*

(I. S2, Z.464-468)

HCSO participants also reported that, by giving them a chance to discuss values and team issues on a different level, the intervention had helped them to deal with their new self-organization structures. The managerial person of the HCSO explained:

*“The effect on the teams throughout was that with the help of these questions they were able to look at how they actually want to perform as a team and what kind of statement they want to make [not only] to the outside world, but also internally.”*

(I. S6, Z. 151-155)

The participants described their experience with the OHL Self-AsseT as contrary to their usual everyday work, where exchanges dealt mainly with organizational matters. Some felt they worked more closely together and had more transparent exchanges. One participant described these changes in teamwork as follows:

*“Before, everyone simply did their job—their clients, their tour. And a lot of things weren’t addressed because there wasn’t enough time or simply: well, that’s it. And now we really focus more on that. Other team members profit too when we talk about it.”*

(I. S3, Z. 502-509)

In contrast, some GPP participants described that they were hardly able to initiate discussions with colleagues. They experienced the process as increasingly difficult and energy-consuming. While this did not prevent them from implementing improvements, they were happy to conclude the project:

*“We didn’t have a doctor with us who participated. And I think that it would have really come out that a part of what was asked in the checklist is done by the doctors. Yes, that also showed me a bit that the exchange [about our practice and ideas to improve] is probably missing a bit.”*

(I. MP3. 1139-1202)

#### 3.5.3. Using a Construction Plan—Making Sense of Ongoing OHL Activities

The participants noted that the meanings and importance of some everyday activities and tasks changed positively through the implementation of the OHL Self-AsseT. This was because, by allocating certain activities to OHL, they developed a broader conceptual understanding of it. To return to the construction metaphor, this conceptual understanding is comparable to a construction plan—an overarching view that helps to prioritize and conduct tasks without getting lost in the work.

Some interviewees explained that discussing OHL led them to recognize the importance of simple patient interventions. For example, several remembered what informational materials were already available and intended to use them more frequently. Others described how, thanks to the intervention, they had connected more with other health professionals and, by doing so, improved care quality and coordination for patients. A participant explained how this worked in her practice:

*“We have taken up a lot more contact with external professionals. And we cooperate with them more often. So that has improved the quality of client care.”*

(I. S5, Z. 536-538)

Some participants described a further link to the concept of OHL through intellectual associations regarding related activities or situations, for example, integrating individual communication techniques such as motivational interviewing into the overall concept. One medical doctor commented:

*“I think that certain terms are now more actively known. That it is not only perceived implicitly, but also explicitly. Because we have often seen that a large part had actually been implemented. But it just never had a title, like a „health literacy Post-It“; but it was just expressed differently and simply done. And now that it is consciously implemented, consciously formulated, I have the impression that we think more about it .”*

(I: FP02, Z.232-240)

Overall, participants considered the intervention as a process of small activities that sparked OHL development or improvement. During the interviews, some coordinating persons mentioned without being asked that they would consider it worthwhile to repeat the OHL Self-AsseT intervention regularly. As one person described:

*“Well, speaking for myself, I see we still have items where we could improve. And I want to remain in a continuous improvement process and not stand still.”*

(MP1, Z. 1029–1032)

## 4. Discussion

Regarding the first implementation of the OHL Self-AsseT, the participants rated the intervention as meaningful and effective. They explained that this was because it offered a practical way to identify and address problems regarding OHL (*Addressing construction sites—building momentum for change*). The experience of being able to initiate change as a group was particularly evident in those teams with greater levels of information exchange. This led to a sense of improved teamwork and efficacy (*Succeeding together in construction—feeling team efficacy during change*). Additionally, using the tool and/or discussing OHL developed participants’ conceptual understanding of OHL. As a result, they gave health literacy-related activities greater priority and importance (*Using a construction plan—making sense of ongoing OHL activities*). Finally, they were convinced that the implementation of the OHL Self-AsseT would lead to improved coordination between health professionals, and enhance care quality. The following paragraphs discuss these main findings both in the light of the participants’ implementation work and regarding the literature.

Theme seven, *Addressing construction sites—building momentum for change*, describes how, on the one hand, the use of the OHL Self-AsseT motivated participants to address changes they had long postponed, and on the other, it helped them to identify and address changes they had not yet envisioned. As described above under *Deciding on implementable OHL measures* such changes included for example the promotion and safeguarding of staff members’ health. This identification of previously unrecognized issues highlights the tool’s potential for OHL improvement. In particular, with repeated application, more issues can be tackled and further aspects of OHL implemented as part of organizational development. This is supported by literature, which approaches OHL improvement as a continuous process [10,30].

In this study participating teams were able to *build a momentum for change*, meaning they managed to move from problem awareness into action. Considering that a failure rate of approximately 70% is commonly cited for organizational change [31], any further scale-up of the OHL Self-AsseT should include an analysis of the factors influencing group activation in relation to the intervention. Evolving theoretical evidence provides some explanations, as it presents constructs and dimensions that positively influence organizational change—particularly the activation of groups toward change.

For example, the theory of organizational readiness for change defines readiness for change as organizational members’ shared commitment to implement a change, i.e., ’change commitment’ [32]. In NPT—the theory that informed the grouping of our themes—within the dimension of ‘coherence’, a group’s ability to make sense of a new practice or intervention is a key mechanism toward change [21]. We describe two themes of implementation work that are in line with these theoretical constructs: First, in “*Promoting health literacy is a responsibility of primary care*”, the participants describe coherent sense-making regarding health literacy, which allows them to see the benefits of strengthening OHL on both the patient-professional and provider levels. Second, in *The OHL Self-AsseT fits with organizational developments*, we showed that the participants already described a high commitment to improving quality—of which, to some extent, they considered OHL an indicator—within their organizations. Therefore, regarding the two theories’ respective definitions, we assume that both the participants’ shared commitment to improving quality of care (including OHL) and their sense-making of OHL as a responsibility of primary care were important factors influencing the groups’ activation toward change.

In view of a scale-up and future uses of the OHL Self-AsseT, we propose using implementation strategies that specifically strengthen sense-making and commitment to the intervention, for example, Weick et al. describe a process of “sense-making with labeling” [33]. This proposition is based on the assumption that the teams used in the reported instance of this initial intervention—the smallest scalable unit—were a positive deviant selection regarding their sense-making and commitment to OHL improvement. Prior to starting the intervention, participating teams should be evaluated for their levels both of sense-making and of commitment; then, any showing lower levels of either should be prepared accordingly.

Theme eight, *Succeeding together in construction—feeling team efficacy during change* describes the team members’ feelings of effectiveness in working together and experiencing success with their chosen improvement measures. Again, theoretical evidence can provide some explanation as to what activated participants toward such positive team perceptions and change. Within Weiner’s theory of organizational readiness for change, accompanying a shared commitment to change, a shared belief in members’ collective capacity to implement a new practice—which Weiner named ‘change efficacy’—is also essential [32]. A very similar concept—‘collective efficacy’—has been derived from the concept of self-efficacy that originated in behavioral change theories [34,35]. Whereas self-efficacy is an important predictor for behavioral change on an individual level, growing evidence indicates that *collective efficacy* impacts change similarly at a group level [36]. Accordingly, this concept will likely become an important predictive outcome parameter of organizational studies, including those in OHL. Naturally, measuring it will require the development and evaluation of specialized instruments [37,38,39,40].

In line with these theories and based on NPT, which highlights the importance of joint action to enact complex interventions in the dimensions of cognitive participation and collective action, our results show that the feeling of team efficacy and a sense of improved teamwork were only apparent in teams that found the time to conduct the assessment and discuss improvement measures in the extremely busy context of everyday practice, despite the COVID-19 pandemic. Implementation work theme three, *Sharing or delegating responsibility for the OHL Self AsseT*, shows that not all self-assessment teams managed to follow the instruction manual, discuss the OHL assessment and decide on improvement measures. Indeed, in teams with insufficient interaction, coordinating persons described only exhaustion—none of the positive team experiences.

This deficiency implies that, for the successful implementation of the OHL Self-AsseT, self-assessment teams must be allocated the time they need to do their work. For some teams, this will require additional implementation strategies from their organizations to support their collaboration. As with any aspect of quality improvement, though, organizational managers and leaders need both to recognize the importance of teamwork as a means of promoting concepts such as OHL and to allocate the necessary resources, which may include implementing effective structures and strategies to support teams [9,24,41]. This need is also well supported by implementation work theme four, *Using supportive structures to carry on with the OHL Self-AsseT*, which describes participants’ support needs regarding orientation and leadership.

The resource investment this entails for managers is worth making, particularly considering that the OHL Self-AsseT’s positive effects on team processes could also be used as building blocks in a multitude of other team development processes and might even be combinable with other team-building approaches. Our results show that the participants of the HCSO could practice some skills (i.e., taking up responsibility for structural changes, as interdisciplinary exchange) they needed for the newly self-organized teams (Buurtzorg model), as well as experienced better teamwork and quality of care [29]. Such considerations are particularly important because insufficient levels of teamwork in health care organizations correlate strongly with high rates of avoidable complications in patients, reduced quality of care, and elevated rates of employee turnover and burnout [42].

Theme nine, *Using a construction plan—making sense of ongoing OHL activities* notes that the newly developed conceptual understanding of OHL has highlighted how even small improvements, such as updating brochures or focusing more on improving communication with patients, are becoming increasingly important for professionals. Our findings also show that, before conducting the intervention, groups were scarcely aware of terms such as HL or OHL, attaching meanings to them very different from those described in this study’s first implementation theme, *Promoting health literacy is as a responsibility of primary care*. For groups who have used the OHL Self-AsseT, this has changed to some degree, as they can now link such terms to practical content. Developing a conceptual understanding of OHL or incorporating practical ideas put forth by a target organization’s members, is an important—though often missing—step toward structural promotion of OHL.

Because of OHL interventions’ complexity, they have to focus on specific organizational contexts to identify capacities, structures and processes that can sustainably support OHL improvement [10]. However, as two recent reviews observe, systematic improvement of organizational and/or individual health literacy is rarely found among health care organizations’ strategic planning priorities [9,43]. This situation must change: only by including OHL in their strategic planning can leaders make meaningful long-term progress in their organizations’ improvement of it.

Compared to the V-HLO-I, which informed the structure of our self-assessment, the OHL Self-AsseT was reported to be relevant and feasible in the primary care setting, as the V-HLO-I is feasible and appropriate in the hospital setting [11].

Methodologically, this qualitative study on the implementation of the OHL Self-AsseT helps us to understand not only the work of individuals and teams involved in its implementation and its impact on their daily practice, but on the value of the intervention process as a demonstration of effective team-building. The inductive and reflexive approach of our reflexive thematic analysis enabled us to identify non-obvious themes running through the team processes. Our NPT-informed structuring of those themes at an advanced stage of the analysis facilitated that process without being too strongly guided by theory. As a subsequent step, a similar process can be used to plan and evaluate the intervention’s scale-up [22,44] alongside quantitative measures such as the Normalisation MeAsure Development questionnaire (NoMAD)—a survey instrument for assessing implementation processes used for complex healthcare interventions [45].

Furthermore, our qualitative data provide new perspectives on the development of OHL in individuals and organizations. While quantitative data can show improvement in measurable ways, these qualitative data highlight the mechanisms through which OHL can be constructed by people and teams. To our knowledge, no previous research has been published on how OHL development takes place—or, in our qualitative paradigm of formulating it, how it is constructed. Still, while these data show initial positive effects in the OHL-Self AsseT’s smallest scalable unit, no conclusion can be drawn regarding its effectiveness at actually improving OHL. Before proceeding to widespread use of the tool, its effectiveness must be tested using appropriate quantitative methods in a larger study group. A further limitation is that we only investigated the perspective of the health professionals, the patient perspective is missing. However, the presented data do allow important conclusions for further development of the tool and its implementation in primary care [13].

## 5. Conclusions

To conclude, the OHL Self-AsseT encouraged teams to initiate change, strengthened teamwork and supported OHL construction in primary care organizations. Especially when the teams were able to implement the OHL Self-AsseT successfully together, this process improved their sense of team efficacy. The initial outcomes described by participants are promising regarding further use and scale-up of the intervention, which will require testing to determine its effectiveness at improving OHL in a later step. In this respect, collective efficacy will likely be useful as a mediating variable and should be developed with this use in mind. Furthermore, in order to strengthen this tool’s implementation and facilitate its scale-up, implementation strategies will have to be developed relating to sense-making, teamwork and compassionate leadership. We are confident that with such strategies, the normalization of repeated iterations of the OHL Self-AsseT intervention will be supported and this finally has the potential to increase and sustain OHL levels in health care organizations.

## Figures and Tables

**Table 1:** Description of sample and data collection.

Time	Sample	Function within the Project	Data Collection
Pre-intervention	4 medical doctors, 2 HCSO managers	manager supporting the project	6 individual interviews
Post-intervention	4 medical doctors 2 HCSO managers 3 managerial persons	manager supporting the project and/or member of the self-assessment team	9 individual interviews
	4 MPAs 3 registered nurses 3 nursing assistants 1 APN	coordinating persons *	3 focus group interviews, 1 individual interview

* Coordinating persons for the OHL Self-AsseT intervention were defined as members of the self-assessment team who took over the coordination of meetings and tasks, including communication with the research team and their managers. Legend: HCSO: home care service organization; MPA: medical practice administrator; APN: advanced practice nurse.

**Table 2:** Description of data analysis along the six phases of reflexive thematic analysis [28].

Phase	Description
1. Familiarizing yourself with the dataset	We transcribed the data verbatim and checked the transcripts against the tapes for accuracy. We familiarized ourselves with the data set by reading and rereading it, as well as by discussing and noting first analytic ideas and upcoming questions. (N.S., D.N., S.M.D.G)
2. Coding	We systematically coded the dataset, first identifying interesting, relevant or meaningful segments, then applying analytically meaningful descriptions (code labels). The initial set of code labels was discussed by N.S., D.N. until they reached a consensus. Later in the process, N.S. coded the data. Codes were checked by D.N.; and coding of challenging parts was discussed in the research group. We gave all data items equal attention. (N.S., D.N., S.M.D.G)
3. Generating themes	Across the dataset we identified themes, which describe a broader, shared meaning than could be conveyed using codes, by compiling clusters of codes that seemed to share core ideas, then collating all data relevant to each candidate theme in an active process. (N.S., D.N., S.M.D.G)
4. Developing and reviewing themes	We organized the candidate themes into thematic maps, then checked across the data set whether the themes highlighted the most relevant patterns (regarding the research question). For each preliminary map, we checked that each theme made sense in relation to the full dataset. To organize the inductively developed themes into a central organizing concept, we used the four NPT dimensions that describe the mechanism of implementation. Themes were checked both against each other and against the original data set until they were internally coherent, consistent, and distinctive. (NS, DN, S.M.D.G.)
5. Refining, defining, and naming themes	We ensured that each theme was clearly demarked and built around a strong core concept—in this case, the NPT mechanism. We gave each theme a concise and informative name. (D.N., N.S.)
6. Writing up	Addressing the research question, we wrote up the analysis in an organized story about the data and topic. We considered that the writing provides an appropriate balance between analytic narrative and illustrative extracts. (D.N., N.S., S.M.D.G., J.G., R.J., A.-S.B.)

**Table 3:** Characteristics of the self-assessment teams.

Organization	Composition of Practice/Nursing Teams	Composition of Self-Assessment Team	Coordinating Person
Medical network of GPPs	13 medical doctors 16 MPA,	4 MPAs	1 MPA
	7 medical doctors, 7 MPA,	1 MPA, 1 medical doctor	1 MPA
	4 medical doctors, 4 MPA,	1 MPA, 1 medical doctor	1 MPA
	7 medical doctors, 7 MPA,	2 MPA, 1 medical doctor	1 MPA
HCSO	4 registered nurses, 10 nursing assistants	4 registered nurses, 10 nursing assistants	2 nursing assistants
	2 registered nurses, 7 nursing assistants	2 registered nurses, 7 nursing assistants	1 registered nurse
	2 registered nurses, 7 nursing assistants	2 registered nurses, 7 nursing assistants	1 nursing assistant
	3 registered nurses, 6 nursing assistants	3 registered nurses, 6 nursing assistants	1 nursing assistant, 1 registered nurse
	3 registered nurses, 10 nursing assistants	3 registered nurses, 10 nursing assistants	1 registered nurse
	5 APNs	5 APNs	1 APN

Legend: GPPs: general practitioners’practices, HCSO: home care service organization, MPA: medical practice administrator, APN: advanced practice nurse.

**Table 4:** Examples of improvement measures.

Health Literacy Dimensions	Assessment Rating: Approval Level (Range)	Documented Improvement Measures	Need For action (Example)	Development Goal (Example)	Improvement Measures (Example)	Quote (Example)
Dimension 1: Provide easy access to primary care service and facilitate navigation within	73.3% (61.9–95.2%)	8	Signs are needed in the underground garage of the building	Patients should quickly see how to get to the practice easily	Signs on doors between the garage and the building	“We instituted a measure by simply putting up a sign in the garage [saying] what floor the practice is on. There were sometimes complaints that they [the patients] had a hard time finding us“ (MP3, Z.157-160)
Dimension 2: Communicating in plain and easy to understand language	74.6% (50.0–95.8%)	5	Oral explanations take too long, patients often have to ask again	Give patients written information	Design patient information flyers	“Well, with fecal test samples that repeatedly caused confusion for the patients, we made a flyer. A colleague found out, that there is a feces sample collector kit. that’s practical for the patients. And we had never even thought about [that]. And then I really noticed: that’s really practical. It’s quicker, too. You can give the flyer to the patient and don’t have to give any long explanations about it” (MP4, Z. 93-102)
Dimension 3: Promoting health literacy of our users	75.3% (50–100%)	5	Patient MPA consultation on changing lifestyle currently not possible due to personnel situation.	Training of new colleagues with Chronic Care Management Module and planning of patient consultation	MPA for consultation hours planned to start in December	“And we’re setting that up now (the consultation), because now we see that with diabetes patients, that interpersonal [counseling] that you can’t achieve in a 15-min consultation, the instructions and of course it’s fun for the MPA, because it’s again more that they can do, working independently” (MP4, Z. 336-343)
Dimension 4: Promoting health literacy of staff members	59.6% (33.3–84.4%)	9	Strengthen knowledge and health literacy of team members.	Every two months one team member prepares an educational training	We do small educational trainings to improve our professional knowledge	“We decided to do team inputs, like, training sessions. And someone from the team has to prepare a topic that is currently of interest” (S3, Z. 102-116).
Dimension 5: Incorporating health literacy into the management and organizational structure	70.3% (56.4–92.6%)	2	Changed procedures are not uniformly implemented	Integrate changes more quickly in practice	1:1 demonstration and control	“Yeah, it always takes some time. Or we give ourselves three weeks, (grins) until something has changed, until routine and the procedure are there. You have to really keep after it actively in the first few days and weeks.” (MPK4, Z. 615-619)
Dimension 6: Promoting health literacy at care interfaces, networks and further activities of the organization	69.0% (50.0–92.6%)	4	Primary care providers do not know one another or allied professionals in their areas	We organize several meetings with other professions, e.g., physiotherapists, and family doctors	We regularly invite allied professionals (physiotherapists, family doctors etc.) in our catchment area to attend our team meetings.	“For example, one team has started to create a network in their area, in their catchment area and include not just the family doctors responsible, but also therapeutic professions. To strengthen interprofessional cooperation. And they regularly invite various family physicians, but also physiotherapists or podiatrists, to their team meetings to promote exchange and so that they can get to know one another, so that cooperation can improve.” (S.5 Z. 192-199)

## Data Availability

The data presented in this study are available on request from the corresponding author. The data are not publicly available to ensure the privacy of our practice partners.
